# Natural History of High-Risk Human Papillomavirus in Kenyan and South African Women: Implications for Vaccination Campaigns and Cervical Cancer Screening Programs

**DOI:** 10.1093/ofid/ofae690

**Published:** 2024-11-22

**Authors:** Anna-Ursula Happel, Elvira Budiawan, Maricianah Onono, Steve Innes, Thesla Palanee-Phillips, Janine Heuvel, Adeebah Rakiep, Sarah Kellow-Webb, Joan Ongere, Imeldah Wakhungu, Zandile Mkhize, La-Donna Kapa, Nompumelelo Sigcu, Smritee Dabee, Gonasagrie Nair, Caitlin Scoville, Nelly R Mugo, Anna-Lise Williamson, Jo-Ann S Passmore, Heather B Jaspan, Renee Heffron

**Affiliations:** Institute of Infectious Disease and Molecular Medicine, University of Cape Town, Cape Town, South Africa; Department of Pathology, University of Cape Town, Cape Town, South Africa; Department of Global Health, University of Washington, Seattle, Washington, USA; Kenya Medical Research Institute, Kisumu, Kenya; Desmond Tutu HIV Centre, University of Cape Town, Cape Town, South Africa; Wits RHI, University of the Witwatersrand, Johannesburg, South Africa; Department of Epidemiology, School of Public Health, University of Washington, Seattle, Washington, USA; Department of Pathology, University of Cape Town, Cape Town, South Africa; Department of Pathology, University of Cape Town, Cape Town, South Africa; Department of Pathology, University of Cape Town, Cape Town, South Africa; Kenya Medical Research Institute, Kisumu, Kenya; Kenya Medical Research Institute, Kisumu, Kenya; Desmond Tutu HIV Centre, University of Cape Town, Cape Town, South Africa; Wits RHI, University of the Witwatersrand, Johannesburg, South Africa; Wits RHI, University of the Witwatersrand, Johannesburg, South Africa; Seattle Children's Research Institute, Seattle, Washington, USA; Desmond Tutu HIV Centre, University of Cape Town, Cape Town, South Africa; Department of Global Health, University of Washington, Seattle, Washington, USA; Department of Global Health, University of Washington, Seattle, Washington, USA; Center for Clinical Research, Kenya Medical Research Institute, Nairobi, Kenya; Institute of Infectious Disease and Molecular Medicine, University of Cape Town, Cape Town, South Africa; Department of Pathology, University of Cape Town, Cape Town, South Africa; Institute of Infectious Disease and Molecular Medicine, University of Cape Town, Cape Town, South Africa; Department of Pathology, University of Cape Town, Cape Town, South Africa; National Health Laboratory Service, Cape Town, South Africa; Institute of Infectious Disease and Molecular Medicine, University of Cape Town, Cape Town, South Africa; Department of Pathology, University of Cape Town, Cape Town, South Africa; Seattle Children's Research Institute, Seattle, Washington, USA; Department of Medicine, University of Alabama at Birmingham, Birmingham, Alabama, USA

**Keywords:** DNA testing, female genital tract, HPV, HPV-35, Papanicolaou smear

## Abstract

**Objective:**

Human papillomavirus (HPV) vaccines and DNA testing roll out in resource-constrained settings. We evaluated the natural history of HPV infections in African women to contribute to normative guidance.

**Methods:**

Women aged 16 to 35 years were enrolled from 3 sites in South Africa and Kenya and followed quarterly for 18 months. A subset was recalled 5 years postenrollment, when Papanicolaou smears were conducted. Endocervical swabs were tested for 36 HPV genotypes by HPV Direct Flow. Logistic regression models identified correlations between demographic, biological, or behavioral factors and baseline high-risk HPV (HR-HPV).

**Results:**

At enrollment, 158 of 311 women (median age, 23 years; IQR, 20–27) had at least 1 HR-HPV genotype. HPV-52 (13.5%), HPV-16 (9.5%), HPV-58 (9.0%), HPV-18 (8.4%), and HPV-35 (8.4%) were most common. Coinfection with low-risk HPVs (odds ratio, 2.65; 95% CI, 1.59–4.45) were associated with HR-HPV positivity, while reported condom use (odds ratio, 0.57; 95% CI, .34–.98) and older age were protective. Of women with HR-HPV at enrollment, 87.3% cleared at least 1 HR-HPV infection over 18 months and 64.6% cleared all such infections. Few (1.9%) had evidence of high-grade cervical abnormalities, among which HPV-35 was the most prevalent during the study.

**Conclusions:**

The high prevalence of HR-HPV emphasizes that HPV vaccination, screening, and testing campaigns in Africa are important. Nonvaccine HPV-35 was as common as HPV-18, suggesting the need to supplement current vaccines with this genotype. HR-HPV clearance was also common, highlighting that clear messaging is needed from health care providers to patients while discussing HPV DNA testing results.

Persistent infections with mucosal types of high-risk (HR) human papillomavirus (HPV) are the causative agents of cervical cancer. Globally, cervical cancer is the fourth-most common cause of cancer incidence and mortality among women, as well as the leading cause of cancer deaths in 42 low- and middle-income countries (LMICs), where 90% of cervical cancer deaths occur [1]. The most common HR-HPV types associated with cervical cancers globally are HPV-16 and HPV-18, with the result that these types have formed the core of prophylactic vaccination development [2]. Most prophylactic HPV vaccines that are licensed globally target these 2 HPV types only (bivalent), 4 HPV types (quadrivalent, including wart-causing HPV-6 and 11), or 9 HPV types (offering additional protection against HPV-31, 33, 45, 52, and 58) [3]. Prophylactic vaccination against HR-HPV types, with 90% coverage by 2030 for girls aged <15 years, is 1 of 3 World Health Organization (WHO) cervical cancer elimination strategies [4]. As of 2022, only 30% of African countries had national HPV vaccination programs [5]. At this rate, it appears unlikely that the WHO 2030 HPV vaccine coverage targets will be met. It therefore remains critical to monitor HR-HPV genotype prevalence in unvaccinated populations to inform HPV vaccination and testing campaigns.

Due to greater sensitivity and cost-effectiveness, the WHO has recommended HPV DNA testing as a first-line screening method for cervical cancer prevention since 2021, starting at 30 years with regular testing every 5 to 10 years [6]. At present, HPV DNA testing is not routinely implemented in national cervical cancer prevention programs in South Africa or Kenya [7, 8], although demonstration programs in South Africa have yielded compelling results [9, 10]. It is thus important to get insights into HR-HPV positivity as determined by DNA testing and its progression to clinical outcomes.

This study aimed to describe the HR-HPV genotype prevalence and subsequent HR-HPV type–specific clearance, persistence, and presence of precancerous cells in unvaccinated South African and Kenyan women to contribute to guidance for the screening and clinical treatment of women with HR-HPV DNA infection who are living in low-resourced settings.

## MATERIALS AND METHODS

### Patient Consent Statement

All women provided written informed consent. The protocol was approved by the Human Subjects Division of the University of Washington (STUDY00000261), Kenya Medical Research Institute Scientific and Ethics Review Unit (SERU/CMR/P0014/3109), University of the Witwatersrand Human Research Ethics Committee (HREC PRC 141112), University of Cape Town Human HREC (HREC 371/2015), and FHI360 (523201).

### Study Cohort

This study was nested within the ECHO Trial (NCT02550067), which enrolled women aged 16 to 35 years desiring effective contraception, to compare HIV incidence rates among women randomized to use a copper intrauterine device, levonorgestrel implant, or intramuscular depot medroxyprogesterone acetate [11]. An ECHO biological mechanisms substudy (ECHO BMS) included 3 of the ECHO Trial sites—Cape Town and Johannesburg, South Africa, and Kisumu, Kenya—where eligible women were offered coenrollment into the parent trial and the substudy. Participants were followed quarterly for up to 18 months, with the final ECHO BMS visit referred to as the “original exit visit,” with extensive cervicovaginal sampling and clinical data collection that concluded in 2018 [12]. Biologic sampling included nurse-collected endocervical swabs, which were archived at −80 °C and used for HPV genotyping in this study.

In 2022, we reopened the substudy cohort and reenrolled contactable and consenting participants who had an endocervical swab collected at enrollment and were available for HPV testing for 1 additional study visit, referred to as the “final exit visit.” At this visit, a pelvic examination, HPV DNA test, and Papanicolaou (Pap) smear were performed. A blood sample was collected for HIV and *Treponema pallidum* (syphilis) testing and urine for pregnancy testing, as well as cervicovaginal fluid for *Chlamydia trachomatis*, *Neisseria gonorrhea*, and bacterial vaginosis (BV) testing. Participants’ demographic, medical, and behavioral information was collected via interview-administered questionnaires.

### HPV Genotyping

All available endocervical swabs were shipped to the University of Cape Town for HPV genotyping. DNA was extracted by the MagNA Pure Compact Nucleic Acid Isolation Kit I (Roche) and tested for the presence of HPV DNA with the HPV Direct Flow Kit (Master Diagnostica), which detects 36 HPV genotypes: 6, 11, 16, 18, 26, 31, 33, 35, 39, 40, 42–45, 51–56, 58, 59, 61, 62, 66–73, 81, 82, 84, and 89. To ensure comparability with other HPV assays, the WHO Global HPV DNA proficiency panel provided by the HPV International Reference Laboratory (Sweden) was run prior to study start.

### Testing for Sexually Transmitted Infections, BV, and Cellular Cervical Changes

Herpes simplex virus 2 serology was performed at the enrollment and original exit visits of the ECHO BMS with HerpeSelect ELISA (Focus Diagnostics). Confirmatory testing was performed via Western blot at the University of Washington [13]. At the enrollment, original, and final exit visits, the GeneXpert Instrument Systems platform (Cepheid Inc) with the Abbott Real Time PCR assay (Abbott Molecular) was used at South African sites to test for *C. trachomatis* and *N. gonorrhea*, and the Panther System (Hologic Inc) was used at the Kenyan site. Treatment was provided for curable sexually transmitted infections diagnosed syndromically or following laboratory testing, according to national guidelines. At enrollment and the final exit visit, BV was assessed by Nugent score at the South African National Institute for Communicable Diseases. Nugent scores 7 to 10 were considered BV positive; 4 to 6, intermediate; and 0 to 3, negative. Symptomatic BV was treated per national guidelines.

Pap smears were conducted at the final exit visit. Samples were examined at local diagnostic laboratories and classified into the following categories via the Bethesda classifications [14]: negative for intraepithelial lesion or malignancy (NILM), low-grade squamous intraepithelial lesion (LSIL), high-grade squamous intraepithelial lesion (HSIL), atypical squamous cells of undetermined significance (ASC-US), atypical squamous cells–cannot exclude high-grade intraepithelial lesion (ASC-H), atypical glandular cells, carcinoma in situ, and invasive cervical cancer. Any abnormal cytology was followed up with appropriate counseling and referral, per the national guidelines.

### Outcomes of Interest

Outcomes of interest were enrollment HPV prevalence, clearance of any or all HR-HPV infections that a woman had at enrollment, redetection of HR-HPV, persistence of HR-HPV infection, and cytologic findings in relation to HR-HPV status.

HR-HPV types were defined in line with the most recent evaluation by the International Agency for Research on Cancer’s Monographs program [15] and included HPV-16, 18, 31, 33, 35, 39, 45, 51, 52, 56, 58, and 59. To increase statistical power, we grouped HPV types based on previously described risk class [16, 17]: HPV-16; HPV-18 and 45; HPV-31, 33, 35, 52, and 58; and HPV-39, 51, 56, and 59. These analyses were limited to women with single infections in the HPV risk groups to not bias time-to-clearance estimates toward certain genotypes.

Clearance was assessed up to 18 months and defined as HR-HPV type–specific positivity at enrollment, followed by 1 or >1 type-specific HPV-negative result at least 3 months after enrollment (primary and supplementary analyses, respectively). Redetection, as a proxy for reinfection or reactivation, was assessed up to 18 months and defined as HR-HPV type–specific positivity at enrollment, followed by at least 2 HR-HPV type–specific negative time points at least 3 months apart and then a type-specific positive result at a later time point. Time to clearance was defined as the time point at which 50% of infections had cleared.

Persistence was assessed up to 18 months and defined as HR-HPV type–specific positivity at enrollment, followed by detection of the same HPV type at an additional visit at least 3 months apart (primary analysis) or 6 months apart (supplementary analysis) without negative testing results in between. Duration of HPV persistence was calculated as the time (days) between the enrollment and the latest visit date when a woman had a HPV DNA–positive result.

In supplementary analyses, we excluded participants with any evidence of high-grade cervical abnormalities (HSIL, ASC-H) to rule out infections that may have already progressed to precancer.

Missing data points were imputed by carrying forward the result from the prior measured time point. Calculations of estimated HR-HPV clearance and persistence were restricted to the subset of women confirmed to be HR-HPV DNA positive for the given HPV type or group of HPV types at enrollment.

### Statistical Analysis

Descriptive analyses were used to present characteristics of the cohort and each outcome of interest. Multivariable logistic regression models assessed factors associated with baseline HR-HPV DNA positivity. We constructed a minimally adjusted model that included country as a confounding factor and a fully adjusted model where all variables were included that individually achieved a *P* value <.20 for the association with the HPV outcome. Time-to-clearance graphs were constructed with the R package *survival* (version 3.7.0) [18]. Analyses were performed in the R framework (version 4.2.2) [19].

## RESULTS

Of the 430 women enrolled in ECHO BMS, 311 from South Africa and Kenya had endocervical swabs collected at enrollment and quarterly that were available for HPV typing and were included in this analysis. At enrollment, the median age was 23 years (IQR, 20–27), 15.5% were infected with *C. trachomatis* and 7.1% with *N. gonorrhea*, 60% were herpes simplex virus 2 seropositive, and 30% had BV (Table 1). A subset of women (155/311, 49.8%) were reenrolled into the study for a final exit visit. The median total follow-up time for these women was 61.5 months (IQR, 60.8–62.7). Slightly more women were reenrolled in South Africa, and there were no demographic differences in the women who reenrolled as compared with ECHO BMS.

**Table 1.** Cohort Characteristics

	Participants, No. (%) or Median [IQR]	
	ECHO BMS^a^ (n = 311)	Reenrolled Cohort (n = 155)
Country		
South Africa	177 (56.9)	71 (45.8)
Kenya	134 (43.1)	84 (54.2)
Marital status		
Married	110 (35.4)	68 (43.9)
Never married	199 (64.0)	86 (55.5)
Previously married	2 (0.6)	1 (0.6)
Education		
None	1 (0.3)	1 (0.6)
Any primary	72 (23.2)	46 (29.7)
Any secondary	216 (69.5)	100 (64.5)
Postsecondary	22 (7.1)	8 (5.2)
No income	255 (82.0)	123 (79.4)
Age at enrollment, y	23 [20–27]	23 [20–27]
Body mass index >30	70 (22.7)	27 (17.6)
Previously pregnant	269 (86.5)	132 (85.2)
No. of living children	1 [1–2]	1 [1–2]
*Neisseria gonorrhoeae*	22 (7.1)	8 (5.2)
*Chlamydia trachomatis*	48 (15.5)	19 (12.3)
Any bacterial sexually transmitted infection	61 (19.7)	26 (16.8)
Bacterial vaginosis: Nugent		
Positive: 7–10	91 (31.3)	36 (25.7)
Intermediate: 4–6	41 (14.1)	20 (14.3)
Negative: 0–3	159 (54.6)	84 (60.0)
Herpes simplex virus 2 serology		
Positive	190 (61.5)	94 (61.0)
Negative	118 (38.2)	59 (38.3)
Equivocal	1 (0.3)	1 (0.6)
Cervical ectopy, %		
None	144 (46.3)	60 (38.7)
1–25	155 (49.8)	91 (58.7)
26–50	9 (2.9)	3 (1.9)
51–75	3 (1.0)	1 (0.6)
Any condomless sex, past 3 mo	213 (68.5)	105 (67.7)
Used condom for last sex act		
No	142 (45.7)	79 (51.0)
Yes	146 (46.9)	62 (40.0)
No sex in the past 3 mo	23 (7.4)	14 (9.0)
Past 3 mo		
Sex for money or gifts	9 (2.9)	3 (1.9)
Sex during a time with vaginal bleeding	44 (14.1)	24 (15.5)
New sex partners	17 (5.2)	9 (5.8)
Multiple sex partners	21 (6.8)	10 (6.5)

^a^Participants from the ECHO Trial biological mechanisms substudy with available swab at enrollment for human papillomavirus testing.

### Baseline HPV Prevalence

**Figure 1.**
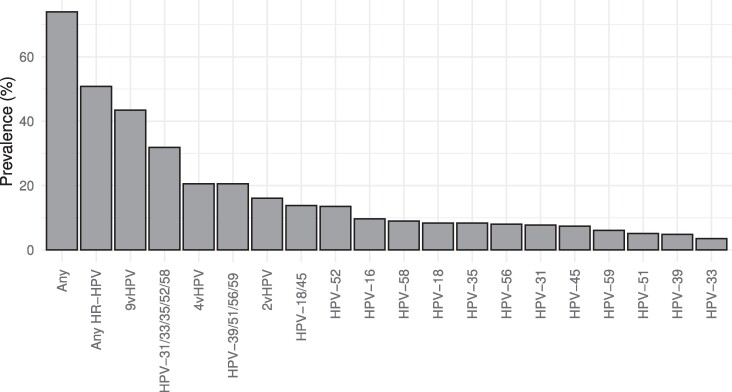
Baseline prevalence of high-risk HPV types in African woman of reproductive age. HPV DNA testing was conducted with endocervical swabs of 311 Kenyan and South African women. 9vHPV: any type targeted by nonavalent HPV vaccine (HPV-6, 11, 16, 18, 31, 33, 45, 52, 58). 4vHPV: any type targeted by quadrivalent HPV vaccine (HPV-6, 11, 16, 18). 2vHPV: any type targeted by bivalent HPV vaccine (HPV-16, 18). Abbreviation: HPV, human papillomavirus.

The HPV prevalence at enrollment was 74.0% (230/311), including 50.8% (158/311) of women who were infected with HR-HPV types (Figure 1). The most common HR-HPV types detected were HPV-52 (prevalence, 13.5%), HPV-16 (9.6%), HPV-58 (9.0%), HPV-18 (8.4%), HPV-35 (8.4%), and HPV-56 (8.0%). Prevalences of previously documented HPV risk classes [16, 17] were as follows: HPV-16 (9.6%); HPV-18 and 45 (13.8%); HPV-31, 33, 35, 52, and 58 (31.8%); and HPV-39, 51, 56, and 59 (19.9%).

At enrollment, HPV types targeted by the bivalent vaccine (HPV-16 and 18) were detected in 16.1% of women; the quadrivalent vaccine (HPV-6, 11, 16, and 18), 20.6%; and the nonavalent vaccine (HPV-6, 11, 16, 18, 31, 33, 45, 52, and 58), 43.4%. HR-HPV types that are not targeted by any of the currently available HPV vaccines (HPV-35, 39, 51, 56, and 59) were observed in 26.0% of the women.

In women who had HPV at enrollment, detection of multiple HPV types was common, with a median of 2 HPV types detected (IQR, 1–4). Similarly, about half of the women with HR-HPV infections had multiple HR-HPV types (Supplementary Table 1).

### Correlates of HR-HPV DNA Positivity at Baseline

Multivariable models showed that women with low-risk HPV infection (odds ratio, 2.65; 95% CI, 1.59–4.45; *P* = .0002) at enrollment were more likely to also have HR-HPV DNA (Supplementary Table 2). Of the demographic factors, older participants were less likely to be infected with HR-HPV at enrollment, with an odds ratio of 0.39 (95% CI, .20–.76; *P* = .006) for participants aged 21 to 24 years as compared with those aged <20 years. Of the behavioral factors, reporting condom use during the last sex act was associated with a reduced risk of HR-HPV infection at enrollment (odds ratio, 0.57; 95% CI, .34–.98; *P* = .035).

### Clearance of HR-HPV Infections

**Figure 2.**
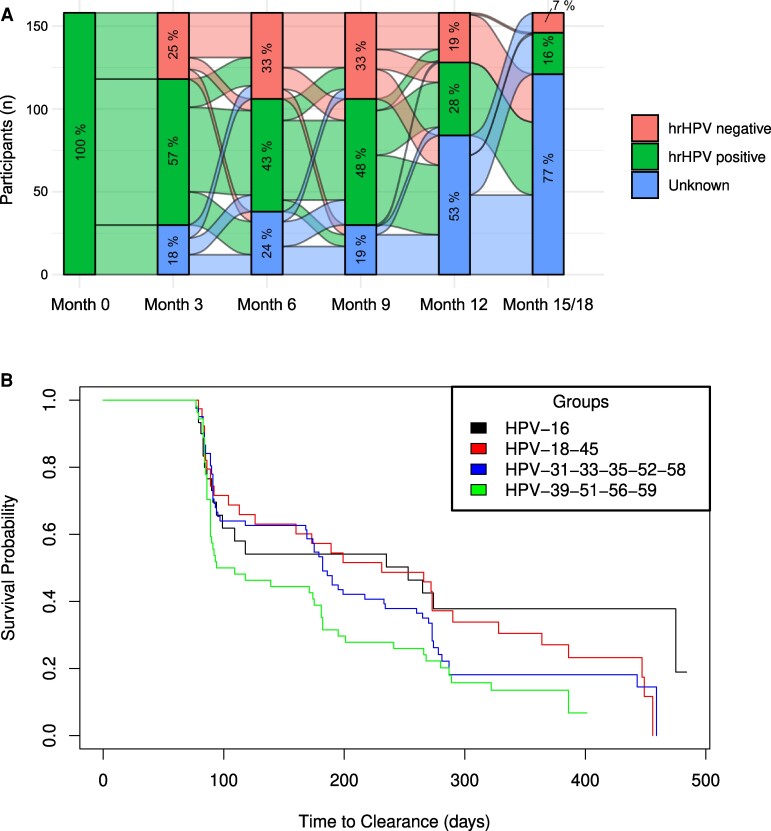
Natural history of HPV infections in African women. *A*, Transitions in HR-HPV infection status over 18 months. Alluvial plot denotes HPV DNA testing results of 153 participants with HR-HPV infections at enrollment. The percentage in each section of the bars indicates the proportion of women with an HR-HPV DNA result that is positive, negative, or unknown. *B*, Time to clearance by HPV risk class over 18 months. Kaplan-Meier plot denotes the time to clearance among women with HPV-16 (n = 30); HPV-18 or 45 (n = 39); HPV-31, 33, 35, 52, or 58 (n = 82); and HPV-39, 51, 56, or 59 (n = 54) at enrollment. Abbreviations: HPV, human papillomavirus; HR, high risk.

Transitions in HR-HPV status were common (Figure 2*A*). One-quarter of participants (38/153) who had HR-HPV at enrollment cleared all their HR-HPV infections by month 3, most of whom remained free of HR-HPV until their final visit. Among participants who were positive for HR-HPV at enrollment, 64.6% cleared all their HR-HPV infections by the time that they reached their original exit visit, and 87.3% cleared at least 1 of their HR-HPV infections over this period (Supplementary Table 3).

In risk group analysis, 60% of women with HPV-16 at enrollment cleared their infection over 18 months, while three-quarters of the women with HPV-18 and 45 (76.9%) and HPV-31, 33, 35, 52, and 58 (74.4%), as well as almost 90% of women with HPV-39, 51, 56, and 59, cleared their infection by month 18 (Figure 2*B*, Supplementary Table 3).

In type-specific analyses, >80% of women who had HPV-18, 39, or 59 at enrollment cleared the infection by the time that they reached the original exit visit, while the observed proportions of women clearing other HR-HPV infections were lower but still >60% (Supplementary Table 3). Similar observations were made when a more stringent definition of clearance was applied that required at least 2 consecutive HR-HPV DNA–negative visits following the HR-HPV DNA–positive visit at enrollment.

Time-to-clearance estimates by HPV risk group (Figure 2*B*) showed that overall the time to clearance differed among women with HPV-16; HPV-18 and 45; HPV-31, 33, 35, 52, and 58; and HPV-39, 51, 56, and 59 (*P* = .04). The median time to clearance was longest for HPV-16 (median, 253 days), followed by HPV-18 and 45 (median, 231); HPV-31, 33, 35, 52, and 58 (median, 182); and HPV-39, 51, 56, and 59 (median, 102). In pairwise comparisons, HPV-16 tended to have a lower probability for clearance as compared with HPV-39, 51, 56, and 59 (adjusted *P* = .093).

In type-specific analyses, the median time to clearance was shortest for HPV-59 (median, 91 days) and longest for HPV-16, 35, and 45 (median, >250 days; Supplementary Table 3).

Similar results were obtained in supplementary analyses that were limited to women who had a Pap smear conducted during the course of the study (n = 155) and excluded women with evidence of high-grade cervical abnormalities (Supplementary Table 3).

### Redetection of HR-HPV Over 18 Months

Cases of redetection—detection of the same HPV genotype after at least 2 HR-HPV type–specific negative time points—were rare and occurred for 5 of the 12 HR-HPV types tested: HPV-18 (1/26 women, 3.8%), HPV-33 (1/1, 9.1%), HPV-52 (5/42, 11.9%), HPV-58 (1/29, 3.4%), and HPV-59 (2/19, 10.5%).

### Persistence of HR-HPV Over 18 Months

In type-specific analyses, HPV-35 had the highest proportion of persistence, with 19 of 26 women (73.1%) having persistent infections, followed by HPV-45 (16/23, 69.6%) and HPV-39 (9/15, 60.0%). Among women with persisting infection, the median time of persistence ranged from <100 days for HPV-18 and 39 to >250 days for HPV-16, 33, 51, 52, and 58 (Supplementary Table 4).

### Cervical Cytology Findings 5 Years Postenrollment versus HR-HPV Infection Status

**Figure 3.**
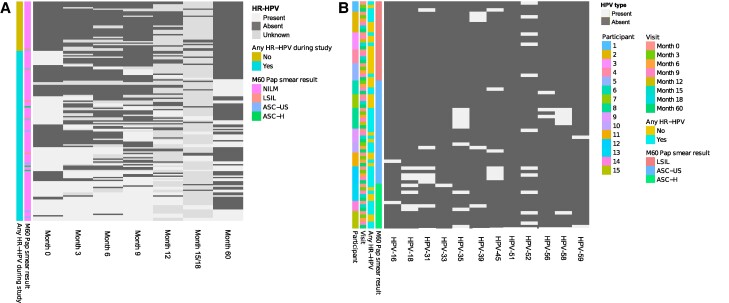
Cervical cytology findings of women 5 years postenrollment as compared with HR-HPV infection status. *A*, Papanicolaou (Pap) smears were conducted for a subset of women reenrolled 5 years after their initial enrollment (n = 155). The heat map shows HR-HPV DNA typing results (light gray, present; dark gray, absent) and is annotated any HR-HPV DNA positivity during the study, with Pap smear result at the final exit visit. *B*, HR-HPV results among 15 women who had Pap smear results other than NILM. The heat map shows HPV type–specific DNA typing results (light gray, present; dark gray, absent) and is annotated by Pap smear category, HR-HPV DNA result, visit, and participant. Abbreviations: ASC-H, atypical squamous cells–cannot exclude high-grade intraepithelial lesion; ASC-US, atypical squamous cells of undetermined significance; HPV, human papillomavirus; HR, high risk; LSIL, low-grade squamous intraepithelial lesion; NILM, negative for intraepithelial lesion or malignancy.

Of the 155 women reenrolled, few (3/155, 1.9%) had evidence of high-grade cervical abnormalities (ASC-H). Other findings included ASC-US (n = 7) and LSIL (n = 5; Figure 3*A*).

Leading up to the ASC-H finding, all 3 women had HR-HPV infections at multiple time points, with at least 4 HR-HPV types detected during follow-up. HPV-35 was detected in all women, alongside HPV-18 and 52 in 2 women (Figure 3*B*). Among the 7 women with ASC-US, 6 had HR-HPV during the study, of which 3 tested HR-HPV DNA positive at all time points evaluated. One woman had only 1 HPV type (HPV-16), while the others had multiple types (range, 2–4). The most commonly detected HR-HPV types among women with ASC-US were HPV-45 (detected in 3 women) and HPV-35, 52, and 56 (detected in 2). Among the 5 women with LSIL, 2 had HR-HPV at most time points evaluated, 2 had HR-HPV at a few time points, and 1 woman did not have any of the HR-HPV types evaluated at any time point. HPV-52 was the most common genotype among women with LSIL, detected in 4 women.

When the analysis was restricted to women with HR-HPV at enrollment who had Pap smear results at the final exit visit (n = 72), 3 (4.2%) had a finding of LSIL, 4 (5.6%) ASC-US, and 3 (4.2%) ASC-H.

Of those who were HR-HPV DNA positive at enrollment with a finding of NILM at the final exit visit, 32.2% (20/62) cleared all HR-HPV infections prior to their original exit visit and remained HR-HPV negative until the final exit visit; 19.35% (12/62) cleared all HR-HPV infections prior to their original exit visit but had HR-HPV at the final exit visit; 11.2% (7/62) still had HR-HPV at the original exit visit but cleared it prior to their final exit visit; a few (4.8%, 3/62) cleared their HR-HPV infections but were HR-HPV positive at their original and final exit visits; and one-third (32.2%, 20/62) had persisting HR-HPV infections over 18 months, of whom 5 had persisting HR-HPV infections until the final exit visit.

## DISCUSSION AND CONCLUSIONS

In this study, which included young women from Eastern and Southern Africa, we found a high prevalence of HR-HPV infections, including those targeted by currently available vaccines, highlighting a substantial public health gap. Nearly 10% of women were infected with HPV-16 at enrollment, which persisted for a median 273 days. These findings on the prevalence and persistence are in line with other studies [20–22]. Given that HPV-16 is one of the most carcinogenic HPV types globally, these results highlight the importance of campaigns in South Africa and Kenya to expand HPV screening and testing to prevent cervical cancer development. We also found a high prevalence of the HPV-35 and HPV-56 HR-HPV types, which are not included in any current vaccines. A high prevalence of HPV-35 has been described among South African women with cervical intraepithelial neoplasia, and while HPV-35 has been found in only 2% of invasive cervical cancers worldwide, it occurs in up to 10% of invasive cervical cancers in sub-Saharan Africa [23–25]. This was confirmed by a recent systematic review that found HPV-35 to have a higher attributable fraction to invasive cervical cancer in Africa (3.6%) than other regions [26]. This warrants further investigations, as these findings suggest that HPV-35 needs to be targeted by future vaccines.

A recent study on the natural history of HPV with 7 years of follow-up found that >90% of HPV infections cleared during the follow-up time frame [16], and our findings were similar. In our study, few women with HR-HPV DNA infections at enrollment had high-grade cytology findings 5 years postenrollment. This has been described [27, 28] and raises the question of how to manage HPV DNA–positive results in resource-constrained settings, many of which are working to adopt the WHO recommendation of DNA testing as the standard screening tool [6]. In a randomized trial setting with data obtained primarily from South Africa, a test-and-treat algorithm to prevent precancer development proved successful [9, 10]. As such, it is crucial that screening, regardless of the test used, be linked to reliable follow-on care, including confirmatory testing and treatment as needed, after implementation on a public health level in LMICs. Among women who cleared their infections, the median time to clearance was type specific and ranged from 3 to 6 months. This has important implications for HPV DNA screening programs, suggesting that at least 6 months should pass prior to repeat testing.

LMICs might face challenges with this approach due to overburdened and underfunded health care systems that struggle to provide access to HPV DNA testing platforms, functional cryotherapy machines, trained providers, and the ability to offer same-day test-and-treatment services [29]. Before population-level implementation in LMICs, careful consideration is needed to guide counseling on HPV DNA results, especially given the high clearance and low progression rates that we and others have documented. The capacity to adequately follow up on women with positive HPV DNA test results is important. It is also important that counseling be informed by evidence in terms of the likelihood that a positive HPV test result will progress to abnormal cytology. Studies are ongoing to optimize scale-up of HPV screen-and-treatment programs in real-world LMIC health care settings, and algorithms that limit treatment to women with certain HR-HPV types and high HPV viral loads seem like a promising approach to implement in LMICs [30, 31].

Our study had some limitations that need to be acknowledged. Women who were enrolled were relatively young (18–35 years), a period associated with less HPV persistence and greater clearance. The exact time to clearance could not be observed, as it occurred within the interval between study visits. Pap smears were not conducted at enrollment, limiting assessment of when the abnormal cytology developed. Strengths include the regular follow-up with frequent sampling over 18 months, use of a gold standard HPV DNA testing method, and follow-up of a subset of women for ∼5 years with clinical outcome measurement.

In conclusion, this study observed a high HR-HPV prevalence in Kenyan and South African women. The high prevalence of HPV types targeted by current HPV vaccines amplify the need to expand ongoing HPV vaccination rollout and screening programs in these countries and their regions. The high prevalence of nonvaccine types HPV-35 and HPV-56 point to the importance of including these types in upcoming vaccine development. The development of precancerous indicators was rare in this low-risk cohort of young women after a HR-HPV DNA–positive test result, emphasizing the recommendation to delay HPV DNA testing to an older age and the need to ensure adequate counseling and follow-up of women after HPV DNA testing in resource-limiting settings to explain results and expected patterns of monitoring in the future.

## Supplementary Data

Supplementary materials are available at *Open Forum Infectious Diseases* online. Consisting of data provided by the authors to benefit the reader, the posted materials are not copyedited and are the sole responsibility of the authors, so questions or comments should be addressed to the corresponding author.

## Supplementary Material

ofae690_Supplementary_Data

## Notes

***Acknowledgments*.** We are grateful to the women who participated in this study and all the research site staff. We thank Sharon Achilles for her scientific input and guidance throughout the project.

***Author contributions.*** R. H., H. B. J., J.-A. S. P., A.-L. W., and N. R. M. conceptualized the HPV substudy to the ECHO Trial. A.-U. H., J. H., A. R., and S. K.-W. performed the experiments. M. O., S. I., T. P.-P., E. B., J. O., I. W., Z. M., L.-D. K., N. S., G. N., C. S., and S. D. provided logistical support and data management and oversaw all data collection. A.-U. H. wrote the first draft of the manuscript. All authors reviewed and approved the final manuscript.

***Data availability statement*.** Data are available in the article.

***Disclaimer.*** The content is the sole responsibility of the authors and does not necessarily represent the official views of the study funders. Under the grant conditions of the Bill & Melinda Gates Foundation, a Creative Commons Attribution 4.0 Generic License has already been assigned to the author accepted manuscript version that might arise from this submission.

***Financial support*.** The ECHO Trial was made possible by the combined generous support of the Bill & Melinda Gates Foundation (OPP1032115), the United States Agency for International Development (AID-OAA-A-15–00045), the Swedish International Development Cooperation Agency (2017/762965-0), the South African Medical Research Council, and the United Nations Population Fund. Contraceptive supplies were donated by the Government of South Africa and the United States Agency for International Development. The ancillary study of biological mechanisms was supported by the National Institute of Child Health and Human Development (5R01HD089831 to R. H. and H. B. J.). The substudy on HPV was supported by the Bill & Melinda Gates Foundation (INV-027876). A.-U. H. receives funding through the EDCTP2 programme (TMA2020CDF-3192-VaViBa) supported by the European Union.

***Potential conflicts of interest*.** All authors: No reported conflicts.
