# Supplementary material for: Natural History of High-Risk Human Papillomavirus in Kenyan and South African Women: Implications for Vaccination Campaigns and Cervical Cancer Screening Programs
Source: Open Forum Infect Dis. 2024 Nov 22;11(12):ofae690. doi: 10.1093/ofid/ofae690 (PMC11631351; doi:10.1093/ofid/ofae690)
Supplement: ofae690_Supplementary_Data [file ofae690_supplementary_data.docx]

| **Supplementary Table 1. Median number of HPV DNA types and prevalence of single and multiple HPV infections at enrollment.** | | | | | | | |
| --- | --- | --- | --- | --- | --- | --- | --- |
| Group | HPV types; median (IRQ) | Max # types | 1 HPV type; n (%) | ≥2 HPV types; n (%) | ≥3 HPV types; n (%) | ≥4 HPV types; n (%) | ≥5 HPV types; n (%) |
| Any HPV, n=230 | 2 (1-4) | 16 | 76 (33.0) | 154 (67.0) | 99 (43.0) | 65 (28.3) | 33 (14.3) |
| Any HR-HPV, n=158 | 1 (1-2) | 8 | 85 (53.8) | 73 (46.2) | 33 (20.9) | 12 (7.6) | 4 (2.5) |

Abbreviations: HPV, human papillomavirus. HR-HPV, high-risk HPV.

| **Supplementary Table 2. Baseline characteristics associated with HR-HPV DNA positivity** | | | | | | | | | |
| --- | --- | --- | --- | --- | --- | --- | --- | --- | --- |
|  | HR-HPV DNA- | HR-HPV DNA+ | Unadjusted | | Minimally adjusted (country) | | Multivariable (country & variables p<0.2 in minimally adjusted model) | | |
|  | N=153 | N=158 | OR (95% CI) | p-value | aOR (95% CI) | p-value | aOR (95% CI) | p-value | |
| Country (%)  Kenya  South Africa | 80 (52.3)  73 (47.7) | 54 (34.2)  104 (65.8) | Ref  **2.11 (1.34, 3.34)** | **0.0001** |  |  |  |  | |
| Not married vs. married (%) | 87 (56.9) | 114 (72.2) | **1.97 (1.23, 3.17)** | **0.005** | 1.34 (0.73, 2.51) | 0.327 |  |  | |
| Education (%)  None / Any primary  Any secondary  Post-secondary | 42 (27.5) 102 (66.7) 9 (5.9) | 31 (19.6) 114 (72.2)  13 (8.2) | Ref  1.51 (0.89, 2.60)  1.96 (0.75, 5.30) | 0.129  0.174 | 0.83 (0.42, 1.63)  1.39 (0.48, 3.68) | 0.592  0.618 |  |  | |
| Earns income vs. not (%) | 32 (20.9) | 24 (15.2) | 0.68 (0.37, 1.21) | 0.191 | 0.86 (0.47, 1.59) | 0.634 |  |  | |
| Age category (%)  16-20  21-24  25-30  31-35 | 35 (22.9) 57 (37.3) 44 (28.8)  17 (11.1) | 50 (31.6) 51 (32.3)  41 (25.9)  16 (10.1) | Ref  0.63 (0.35, 1.11)  0.65 (0.35, 1.19)  0.66 (0.29, 1.48) | 0.110  0.167  0.311 | **0.50 (0.27, 0.90)**  0.64 (0.29, 1.02)  **0.42 (0.18, 1.00)** | **0.023**  0.060  **0.050** | **0.39 (0.20, 0.76)**  **0.49 (0.24, 0.98)**  **0.43 (0.17, 1.05)** | **0.006**  **0.046**  **0.065** | |
| BMI >30 vs. ≤30 (%) | 34 (22.4) | 36 (22.9) | 1.03 (0.61, 1.76) | 0.906 | 0.72 (0.40, 1.28) | 0.258 |  |  | |
| Previously pregnant vs. never been pregnant (%) | 138 (90.2) | 131 (82.9) | 0.54 (0.26, 1.02) | 0.063 | 0.90 (0.42, 1.86) | 0.406 |  |  | |
| *N. gonorrhoeae* DNA positive vs. negative (%) | 8 (5.2) | 14 (9.0) | 1.79 (0.74, 4.60) | 0.206 | 1.44 (0.59, 3.76) | 0.436 |  |  | |
| *C. trachomatis* DNA positive vs. negative (%) | 22 (14.4) | 26 (16.7) | 1.19 (0.64, 2.22) | 0.579 | 1.06 (0.56, 2.00) | 0.862 |  |  | |
| Bacterial vaginosis (%)  Negative  Intermediate  Positive | 80 (56.7) 21 (14.9)  40 (28.4) | 79 (52.7) 20 (13.3)  51 (34.0) | Ref  0.96 (0.48, 1.92)  1.29 (0.77, 2.17) | 0.918  0.333 | 0.96 (0.47, 1.93)  1.20 (0.71, 2.03) | 0.914  0.497 |  |  | |
| HSV-2 serology positive vs. negative (%) | 93 (61.2) | 97 (62.2) | 1.04 (0.66, 1.65) | 0.857 | 0.99 (0.62, 1.58) | 0.963 |  |  | |
| LR-HPV DNA positive vs. negative (%) | 65 (42.5) | 105 (66.5) | **2.68 (1.70, 4.27)** | **< 0.0001** | **2.36 (1.47, 3.81)** | **0.0004** | **2.65 (1.59, 4.45)** | **0.0002** | |
| Cervical ectopy (%)  None  1-25%  26-50%  51-75% | 58 (37.9) 88 (57.5) 5 (3.3)  2 (1.3) | 86 (54.4)  67 (42.4) 4 (2.5)  1 (0.6) | Ref  0.51 (0.32, 0.79)  0.54 (0.19, 4.90)  0.38 (0.01, 2.67) | **0.005**  0.372  0.379 | 0.83 (0.40, 1.72)  0.74 (0.17, 3.14)  0.33 (0.02, 3.54) | 0.612  0.683  0.372 |  |  | |
| Any condomless sex (past 3 months) vs. none reported (%) | 100 (65.4) | 113 (71.5) | 1.33 (0.82, 2.16) | 0.243 | 1.22 (0.75, 1.99) | 0.428 |  |  | |
| Used condom for last sex act vs. none reported (%)^#^ | 75 (53.2) | 71 (48.3) | 0.82 (0.51, 1.31) | 0.407 | 0.69 (0.42, 1.12) | 0.132 | **0.57 (0.34, 0.98)** | **0.035** |  |
| New sex partners past 3 months vs. none reported^#^ (%) | 6 (4.3) | 9 (6.2) | 1.47 (0.52, 4.51) | 0.470 | 1.22 (0.42, 3.77) | 0.722 |  |  | |
| Multiple sex partners past 3 months vs. none reported (%)^#^ | 8 (5.7) | 12 (8.2) | 1.47 (0.59, 3.88) | 0.408 | 1.18 (0.46, 3.14) | 0.734 |  |  | |

^#^Analyses exclude n=23 women who reported to not have had any sex in the past 3 months. Abbreviations: HPV, human papillomavirus. HR-HPV, high-risk HPV. LR-HPV, low-risk HPV. OR, odds ratio. aOR, adjusted OR.

| **Supplementary Table 3. Type-specific clearance or HR-HPV over up to 18 months.** | | | | | |  |
| --- | --- | --- | --- | --- | --- | --- |
|  | All women | | | Excluding women with evidence of high-grade cervical abnormalities [HSIL, ASC-H] during study | | |
| HPV type | n/N (%) of women who cleared infections, lenient | n/N (%) of women who cleared infections, stringent | Time to clearance, days, median (IRQ) ^#^ | n/N (%) of women who cleared infections, lenient | Time to clearance, days, median (IRQ) ^#^ |  |
| Any HR-HPV | 138/158 (87.3) | 108/138 (78.3) | 160 (97 – 182) | 57/65 (87.7) | 175 (118 - 199) |  |
| All HR-HPV | 102/158 (64.6) | 74/138 (53.6) | 260 (190 – 281) | 47/65 (72.3) | 199 (175 – 287) |  |
| HPV-18 and 45 | 30/39 (76.9) | 22/35 (62.9) | 231 (126 - 364) | 13/14 (92.9) | 150 (92 – NA) |  |
| HPV-31, 33, 35, 52, and 58 | 61/82 (74.4) | 48/79 (60.8) | 182 (169 - 260) | 25/33 (75.8) | 195 (175 – 287) |  |
| HPV-39, 51, 56, and 59 | 47/54 (87.0) | 41/53 (77.4) | 102 (89 - 182) | 23/27 (85.2) | 118 (89 – 268) |  |
| HPV-16 | 18/30 (60.0) | 16/29 (55.2) | 253 (99 - NA) | 4/5 (80.0) | 86 (83 – NA)_ |  |
| HPV-18 | 21/26 (80.9) | 14/23 (60.9) | 173 (104 – 364 ) | 10/10 (100) | 102 (89 – NA) |  |
| HPV-31 | 19/24 (79.2) | 11/19 (57.9) | 182 (92 - NA) | 7/7 (100) | 175 (92 – NA) |  |
| HPV-33 | 7/11 (63.6) | 7/11 (63.6) | 217 (89 – NA) | 0/1 (0) | NA |  |
| HPV-35 | 18/26 (69.2) | 14/24 (58.3) | 273 (182 - NA) | 8/10 (80.0) | 278 (168 – NA) |  |
| HPV-39 | 13/15 (86.7) | 9/11 (81.8) | 181 (89 – NA) | 6/6 (100) | 222 (89 – NA) |  |
| HPV-45 | 15/23 (65.2) | 12/21 (57.1) | 287 (272 – NA) | 4/6 (66.7) | 282 (199 – NA) |  |
| HPV-51 | 12/16 (75.0) | 9/14 (64.3) | 182 (89 – NA) | 2/4 (50.0) | 182 (182 – NA) |  |
| HPV-52 | 32/42 (76.2) | 24/39 (61.5) | 118 (91 – 273 ) | 11/17 (64.7) | 199 (91 – NA) |  |
| HPV-56 | 19/25 (76.0) | 16/24 (66.7) | 181 (118 – 322 ) | 11/13 (84.6) | 139 (89 – NA) |  |
| HPV-58 | 19/28 (67.9) | 15/26 (57.7) | 182 (97 – NA) | 5/9 (55.6) | 364 (190 – NA) |  |
| HPV-59 | 17/19 (89.5) | 14/16 (87.5) | 91 (89 – 280 ) | 8/10 (80.0) | 90 (85 – NA) |  |
| Each analysis included women who were DNA+ for a given HR-HPV type at enrollment. The lenient definition of clearance required a woman to have at least one HR-HPV DNA- time point after enrollment, while the stringent definition required at least two HR-HPV DNA- time points after enrollment without a positive test in-between. N indicates the number of women include in each analysis. ‘Any HR-HPV’ required a woman to have cleared at least one of the HR-HPV infections present, while ‘all HR-HPV’ required a woman to have cleared all HR-HPV infections. For HPV risk groups (HPV-18 and 45, HPV-31, 33, 35, 52 and 58, and HPV-39, 51, 56 and 59) the analysis was limited to women with single HPV type infections ^#^Time point at which 50% of infections had cleared, lenient definition. Abbreviations: HPV, human papillomavirus. HR-HPV, high-risk HPV. HSIL, high-grade squamous intraepithelial lesion . ASC-H, atypical squamous cells– cannot exclude high-grade intraepithelial lesion. | | | | | | |

| **Supplementary Table 4. Type-specific persistence or HR-HPV over 18 months.** | | | |
| --- | --- | --- | --- |
| HPV type | n/N (%) of women with persistent infections, lenient | n/N (%) of women with persistent infections, stringent | Duration of persistence, days, median (IRQ) ^#^ |
| HPV-16 | 15/30 (50.0) | 11/27 (40.7) | 273 (93-388) |
| HPV-18 | 11/26 (42.3) | 5/24 (20.8) | 91 (89-274) |
| HPV-31 | 14/24 (58.3) | 9/22 (40.9) | 192 (93-271) |
| HPV-33 | 5/11 (45.5) | 4/11 (36.4) | 273 (132-444) |
| HPV-35 | 19/26 (73.1) | 10/24 (41.7) | 168 (91-286) |
| HPV-39 | 9/15 (60.0) | 3/13 (23.1) | 96 (89-182) |
| HPV-45 | 16/23 (69.6) | 10/21 (47.6) | 182 (92-344) |
| HPV-51 | 5/16 (31.2) | 4/16 (25.0) | 360 (187-390) |
| HPV-52 | 15/42 (35.7) | 12/40 (30.0) | 273 (180-369) |
| HPV-56 | 11/25 (44.4) | 6/24 (25.0) | 182 (91-314) |
| HPV-58 | 14/28 (50.0) | 9/25 (36.0) | 264 (91-385) |
| HPV-59 | 5/19 (26.3) | 4/18 (22.2) | 198 (94-354) |
| Each analysis included women who were DNA-positive for a given HR-HPV type at enrollment. The lenient definition of persistence required a woman test HR-HPV DNA-positive for more than one consecutive visit after enrollment at least 3 months apart, while the stringent definition required detection of HR-HPV type at two consecutive time points at least 6 months apart without a negative test in-between. N indicates the number of women include in each analysis. ^#^Calculated including only women who had persistent infections (lenient definition). Abbreviations: HPV, human papillomavirus. HR-HPV, high-risk HPV. | | | |
